# Effectiveness of fissure sealants following different silver fluoride application protocols in MIH-affected molars: randomized clinical study

**DOI:** 10.1007/s00784-026-06856-1

**Published:** 2026-03-31

**Authors:** Nagihan Cayiroglu, Elif Ballikaya, Gizem Erbas Unverdi, Zafer Cavit Cehreli

**Affiliations:** 1 Department of Pediatric Dentistry, Faculty of Dentistry, Hacettepe University, Ankara, 06100 Turkey; 2 Department of Pediatric Dentistry, Faculty of Dentistry, Istanbul Health and Technology University, Istanbul, Turkey

**Keywords:** Molar incisor hypomineralization, Silver fluoride, Resin-based fissure sealant, Glass ionomer fissure sealant

## Abstract

**Objectives:**

To compare the clinical efficacy of glass ionomer sealants (GIS) and resin-based sealants (RBS) applied immediately or one week after silver fluoride (SF) application to permanent molars affected by molar incisor hypomineralization (MIH) in children at high risk for caries.

**Methods:**

A total of 216 MIH-affected molars (ICDAS 1–2) from 102 children aged 6–14 years were randomly allocated to six groups: Resin- or glass ionomer–based fissure sealants were placed either one week after the application of silver fluoride or during the same clinical session. In the same-visit protocols, sealants were applied immediately after silver fluoride treatment, either following gentle blot drying with cotton or after immediate rinsing. Hypersensitivity, caries progression, and sealant retention were assessed at 1, 6, and 12 months. Hypersensitivity was evaluated using the Schiff Cold Sensitivity Scale (SCASS). Sealant performance was analyzed using modified USPHS criteria. Statistical analyses included Fisher’s Exact Test and the Friedman test.

**Results:**

Full retention was achieved in all resin-based sealant groups, while the lowest retention rate (88.9%) was recorded in the glass ionomer sealant group applied one week after silver fluoride. Caries occurred in 2.8% of teeth and was confined to three groups: glass ionomer sealants placed one week after silver fluoride, resin-based sealants applied immediately following blot drying, and glass ionomer sealants applied immediately after rinsing. All other groups showed complete clinical success. No statistically significant differences were found among groups regarding caries prevention (*p* = 1.000) or sealant retention (*p* = 0.062). Hypersensitivity decreased significantly over time in all groups.

**Conclusion:**

Resin-based and glass ionomer fissure sealants placed either one week after silver fluoride application or immediately following treatment, using rinsing or blot-drying protocols, showed similar outcomes over a 12-month follow-up period in terms of caries prevention and reduction of hypersensitivity. Nonetheless, within the limitations of this study, sealants placed immediately following silver fluoride application demonstrated a greater frequency of discoloration.

**Clinical significance:**

Both glass ionomer and resin-based fissure sealants can be successfully applied either immediately or one week following silver fluoride treatment to provide protection for MIH-affected permanent molars.

**Clinical trials registration number:**

NCT06641011.

## Introduction

Molar incisor hypomineralization (MIH) is a developmental enamel defect that affects one to four first permanent molars, often with incisors, and appears as white-yellow or brown opacities that may lead to enamel breakdown in severe cases [1, 2]. Its global burden is considerable, affecting approximately 878 million people worldwide and leading to about 17.5 million new cases each year [3].

Managing MIH is challenging due to its diverse clinical outcomes and the heightened sensitivity of affected teeth [4]. The weakened enamel is highly prone to post-eruptive breakdown, increasing the risk of caries and causing discomfort during everyday oral functions [5]. The primary goals of treatment are to reduce sensitivity, preserve remaining enamel, and improve aesthetics [6]. Management should be tailored to the severity of the condition and the individual needs of the patient [7]. Consequently, treatment is often intensive and can markedly affect a child’s quality of life as well as their psychosocial well-being [3].

Early diagnosis and preventive strategies are essential to minimize complications of MIH and ensure effective care. Minimally invasive techniques are recommended to prevent dental caries and hypersensitivity in hypomineralized molars [8]. Preventive approaches generally involve thorough oral hygiene guidance, regular use of fluoride toothpaste, and application of topical fluoride varnish, along with frequent follow-up visits [9].

To manage hypersensitivity, several remineralizing and desensitizing agents have been suggested. Among these, casein phosphopeptide-amorphous calcium phosphate (CPP-ACP) has shown superior efficacy compared to conventional fluoride toothpastes [10]. Silver diamine fluoride (SDF) is another non-invasive treatment option that occludes dentinal tubules and enhances mineral density [11]. Its clinical use, however, can be restricted by gingival irritation and by the unfavorable taste and odor linked to its alkaline, ammonia-containing formulation. Water-based silver fluoride (SF) has been proposed as an alternative to overcome these limitations; however, the available evidence regarding its equivalence to SDF is scarce and largely based on data provided by the manufacturer. According to the manufacturer, it provides comparable effects while maintaining a neutral pH (7.4) and potentially improved patient acceptance [12].

As a first-line preventive approach, fissure sealants are considered an effective, accessible, and practical method for controlling occlusal caries. They promote enamel remineralization, inhibit biofilm accumulation, and create a surface that is easier to clean [13, 14]. As a comprehensive guideline, the Würzburg Concept 2.0 [15] recommends sealant application as an initial treatment to reduce hypersensitivity in molars affected by MIH, regardless of whether enamel breakdown is present.

SDF has also shown effectiveness in reducing hypersensitivity of MIH affected molars, either alone or in combination with atraumatic restorative treatment (ART) sealants-an approach commonly-known as the silver-modified atraumatic restorative technique (SMART) [16]. High-viscosity glass ionomer cements (HVGICs) used in SMART sealants provide good handling and mechanical characteristics; however, their retention decreases over time, with only about 66.6% of restorations retaining after three years [17].

Evidence on how SDF influences the retention and adhesion of restorative materials remains inconclusive [18]. While a meta-analysis of several in vitro studies indicates that SDF does not compromise the bond strength of glass ionomer cement to dentin, it may weaken the adhesion in resin-based systems [18]. Postponing the bonding procedure for one week after SDF application has been proposed as a way to reduce this negative impact [19].

So far, no clinical research has directly compared two non-invasive preventive strategies using silver fluoride in combination with either resin-based or glass ionomer fissure sealants, applied under varying timing and handling protocols in teeth affected by MIH. Accordingly, this study sought to assess the clinical effectiveness of both sealant materials when placed either immediately or one week after SF application on MIH-affected permanent molars in patients at high risk for caries. In addition, the study examined how different handling protocols; specifically, rinsing versus blot drying the SF influence the clinical success of fissure sealants applied immediately after SF treatment. We tested the null hypotheses that there would be no differences between the groups in sealant retention and in the incidence of secondary caries over the follow-up period.

## Materials and methods

A randomized controlled clinical trial was conducted following approval of the Local Ethics Committee (Reg. no: KA-23037, ClinicalTrials.gov NCT06641011). Informed consent was obtained from all parents, and the study adhered to the CONSORT guidelines [20].

### Selection of participants

The study enrolled children attending the Pediatric Dentistry Clinic at the Hacettepe University School of Dentistry for routine dental examinations and care. A total of 216 MIH-affected first permanent molars from 102 healthy children aged 6–14 years were included.

Inclusion criteria were as follows:

- Presence of at least one fully erupted permanent first molar diagnosed with MIH according to the criteria of European Academy of Pediatric Dentistry (EAPD) [21]
- Enamel defects of occlusal fissures scored as 1 or 2 according to the International Caries Detection and Assessment System (ICDAS II)
- MIH-affected molars with opacities measuring ≥ 2mm on the occlusal surface and involving at least one additional surface
- Patients and parents who have agreed to participate and signed the informed consent form

Exclusion criteria included:

- The presence of syndromes associated with enamel malformations,
- Poor patient cooperation,
- Systemic conditions requiring continuous medication,
- Ongoing orthodontic treatment,
- Teeth classified as ICDAS 3–6, or presenting with existing restorations, fluorosis, or pulpal symptoms.

As no previous studies compared the retention of resin-based and glass-ionomer sealants following SF application in MIH-affected molars, the sample size estimation was based on a previous study [22] that compared these sealant materials in healthy molars. The calculation was performed using G-power (version 3.1.9.4) for a two-tailed hypothesis with a 5% margin of error and 80% power (z test, differences between percentages in independent groups), resulting in a required sample size of 33 teeth per group. Considering a potential dropout rate of 10%, 36 teeth were included in each group, resulting in a total of 216 teeth across all subgroups.

### Study design

Teeth meeting the inclusion criteria were randomly assigned to six groups using a computer-generated randomization sequence (www.random.org). Allocation concealment was maintained through sequentially numbered, opaque, sealed envelopes prepared by an independent investigator which were opened only at the time of fissure sealant placement.

Group 1: Resin-based fissure sealant (RBS; 3 M™ Clinpro™ Sealant, 3 M ESPE, St. Paul, MN, USA), applied one week after SF application.

Group 2: Glass ionomer fissure sealant (GIS; GC Fuji TRIAGE^®^, GC Europe N.V., Leuven, Belgium), applied one week after SF application.

Group 3: RBS applied immediately after SF application following blot-drying.

Group 4: GIS applied immediately after SF application following blot-drying.

Group 5: RBS applied immediately after SF application following rinsing.

Group 6: GIS applied immediately after SF application following rinsing.

### Baseline assessments

Prior to treatment, initial plaque accumulation and gingival health of the teeth were assessed using the Silness and Löe Plaque Index and the Löe and Silness Gingival Index, respectively [23, 24]. DMFT/dmft scores (decayed, missing and filled teeth) were recorded for each patient according to the criteria established by the World Health Organization (WHO). The teeth were thoroughly dried, and enamel surfaces were evaluated according to the ICDAS II criteria. Tooth sensitivity was assessed using the Schiff Cold Air Sensitivity Scale (SCASS) [25], where 0 indicates no response, 1 indicates reported discomfort without physical reaction, 2 indicates withdrawal from the stimulus; and 3 reflects both withdrawal and a request to stop the stimulus. Primary contact points with opposing teeth were identified using articulating paper, and baseline photographs were taken. All treatments were performed by a single operator.

### Clinical procedures

Tooth surfaces were cleaned using a slow-speed polishing brush, rinsed with an air-water spray, and air-dried. Clinical procedures were then performed according to the groups (*n* = 36 teeth per group) described in Section Study design.

In all groups, an SF solution (Lot No: 1137328) was applied to the entire tooth surface with a microbrush and allowed to remain for 60 s. For groups 1 and 2, patients returned after one week, when hypersensitivity was reevaluated and photographs were obtained to record any possible discoloration associated with the SF application. Dental plaque was removed using a slow-speed polishing brush under water cooling. In the RBS groups, teeth were isolated with cotton rolls and 32% phosphoric acid gel (3 M™ ScotchbondTM Universal Etchant, 3 M ESPE, Germany) was applied on the pits and fissures for 30 s. The etchant was rinsed off with water for 20 s and the enamel was thoroughly air-dried.

A universal adhesive (3 M™ Scotchbond Universal™, 3 M/ESPE, Germany) was then applied and air-thinned for at least 5 s using an air spray held approximately 1 cm from the tooth. The adhesive was subsequently light-cured for 10 s with a new LED curing unit (GC D-light Pro). The RBS was applied using its applicator, evenly distributed over the pits and fissures and any air bubbles were removed. The material was light-cured for 20 s on both occlusal and buccal/palatal surfaces. The surface was checked with an explorer to confirm proper adaptation. The oxygen inhibition layer was removed using a damp cotton pellet. Occlusal contacts were evaluated using articulating paper, and any required adjustments were made using finishing burs followed by polishing with a rubber cup.

In group 2, at the one-week follow-up visit, the teeth were isolated with cotton roll. A 20% polyacrylic acid conditioner (GC Cavity Conditioner, GC Europe N.V., Leuven, Belgium) was applied to the pits and fissures for 10 s, then rinsed off with water and air dried. GIC was prepared in a capsule mixer at standard speed for 10 s and placed using a gun applicator. The tooth remained isolated for 2 min to allow setting of the material. Occlusal contacts were assessed with articulating paper, and any necessary adjustments were carried out using water-cooled, low-speed aluminum oxide discs followed by polishing with a rubber cup.

For sealants placed immediately after SF treatment using blot drying (groups 3 and 4), the SF was applied and left undisturbed for one minute. The solution was then gently removed from the fissures with a cotton pellet while maintaining cotton roll isolation. Sealant placement was subsequently carried out according to the same protocols used for the glass ionomer and resin-based sealants. For sealants placed immediately after SF treatment with rinsing (Groups 5 and 6), the SF was applied and allowed to act for one minute before being rinsed off with water for 5 s. Sealant placement was then completed according to the previously described protocols.

**Fig. 1:**
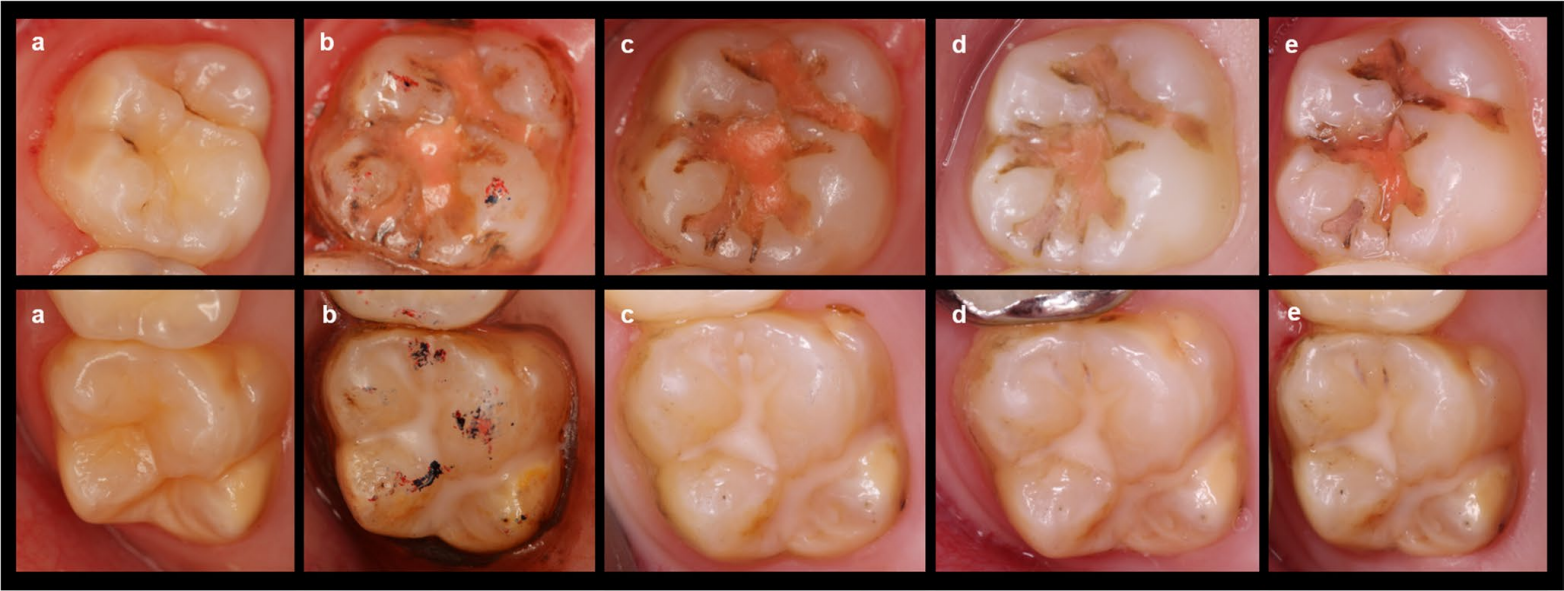
Clinical intra-oral photographs of teeth with resin-based and glass ionomer sealants at all time points. **a**: baseline, **b**: post-op, **c**: 1 month **d**: 6 months, **e**: 12 months

Following the completion of SF and fissure sealant applications, patients were recalled for follow-up visits at 1, 6, and 12 months. In accordance with SF application guidelines, SF was re-applied biannually, at the 6- and 12-month visits. At each follow-up visit, hypersensitivity was reevaluated before SF was reapplied. Clinical assessments included evaluation of caries development, integrity of fissure sealants, and tooth sensitivity, as well as plaque accumulation and gingival health around the treated tooth. Intra-oral photographs were obtained at baseline, immediately after treatment and during recalls to evaluate discoloration, secondary caries and sealant loss under magnification (Fig. 1). The United States Public Health Service (USPHS) criteria (anatomical form, marginal adaptation, surface roughness, marginal discoloration and secondary caries) were used for clinical evaluation of fissure sealants.

### Statistical analysis

Data were analyzed using the IBM SPSS Statics software V23. Survival analyses for caries prevention and material retention, derived from clinical evaluation data, were conducted using the Kaplan–Meier method. The significance of differences in mean age between groups were evaluated using Student’s t-test. Categorical outcomes were analyzed using Fisher’s exact test for between-group comparisons, while Cochran’s Q test, followed by Bonferroni-corrected McNemar tests, was applied for within-group longitudinal comparisons. For the SCASS measurements, which represent a numerical outcome variable, the data were reanalyzed using generalized estimating equations (GEE) with an exchangeable working correlation structure. Patient ID was specified as the clustering variable in order to account for the correlation among teeth belonging to the same patient. When a significant overall group effect was detected, pairwise comparisons between groups were performed using estimated marginal means with Bonferroni correction.

All patient treatments were performed by a single clinician, while clinical evaluations were independently performed by two different clinicians simultaneously. In cases of disagreement, a senior clinician was consulted to reach a consensus. To assess intra- and inter-examiner reliability in evaluating sealant retention, 25 patients were re-examined after a 7-day interval, and Cohen’s kappa statistic were evaluated. The intra-examiner reliability values were 0.91 and 0.93, while inter-examiner reliability was 0.91. A p-value < 0.05 was considered statistically significant for all analyses.

## Results

**Fig. 2:**
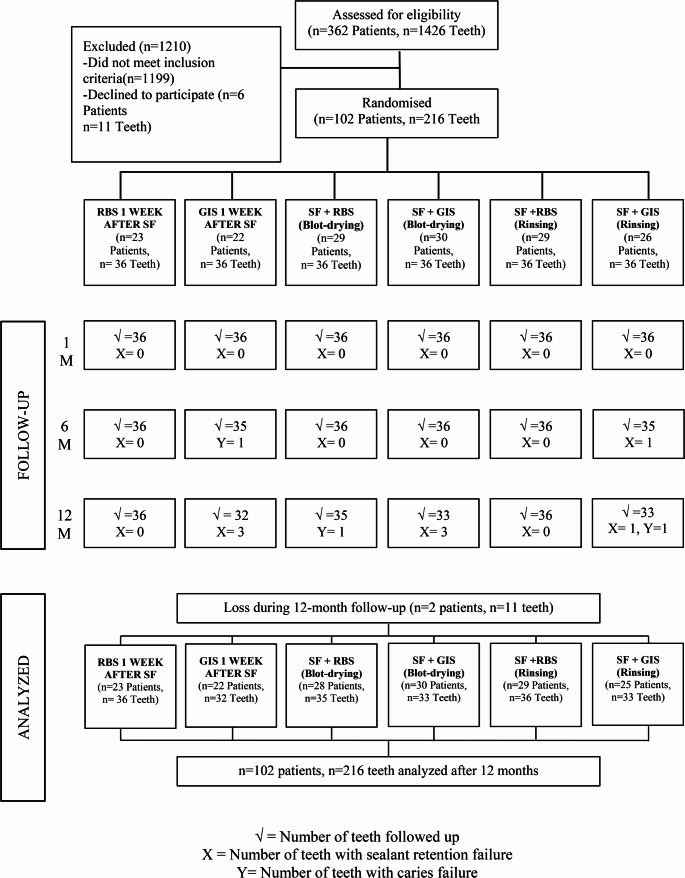
Study flow diagram illustrating participant recruitment, tooth allocation and follow up over a 12-month period

A total of 102 patients (52% girls and 48% boys) with a mean age of 9.06 ± 1.92 years were included in the study. A total of 216 teeth, with 36 teeth in each group, were evaluated over the 12-month follow-up. The recruitment and flow diagram of patients is presented in Fig. 2. The mean dmft and DMFT scores of all patients was 3.25 ± 3.05 and 1.70 ± 1.58, respectively.

Table 1 shows a summary of clinical evaluations using the modified USPHS criteria and retention rates. The retention of all 216 sealants was assessed both collectively and by individual group. Overall, 95.8% of the restorations (n = 207) were successful, whereas 4.2% (n = 9) were unsuccessful. When evaluated by groups, all sealants in Groups 1, 3, and 5 (resin-based fissure sealant groups) demonstrated 100% success. In Group 2, the success rate was 88.9% (n=32) with a failure rate of 11.1% (n=4); in Group 4, the success rate was 94.4% (n=34) and the failure rate was 5.6% (n=2); and in Group 6, the success rate was 91.7% (n=33) with a failure rate of 8.3% (n=3). No significant difference was found between the groups (p=0.062) (Table 2).

Caries progression was monitored across all groups. No caries was detected in 98.6% of the teeth (*n* = 213), while lesions were identified in 1.4% (*n* = 3). Likewise, there were no statistically significant differences in caries occurrence among the groups (*p* = 1.000) (Table 2).

**Table 1:** Clinical performance of sealants according to the modified USPHS criteria for occlusal surfaces. A= Alfa, B= Bravo, C= Charlie

Time	Group 1			Group 2			Group 3			Group 4			Group 5			Group 6		
	1	6	12	1	6	12	1	6	12	1	6	12	1	6	12	1	6	12
Score	n(%)	n(%)	n(%)	n(%)	n(%)	n(%)	n(%)	n(%)	n(%)	n(%)	n(%)	n(%)	n(%)	n(%)	n(%)	n(%)	n(%)	n(%)
Anatomical form																		
- A	36(100)	34(94.4)	30(83.3)	35(97.2)	19(52.8)	17(48.6)*	35(97.2)	32(88.9)	27(75)	28(77.8)	18(50)	11(30.6)	36(100)	33(91.7)	24(66.7)	35(97.2)	21(58.3)	16(45.7)*
- B	0(0)	2(5.6)	6(16.7)	1 (2.8)	16(44.4)	15(42.9)*	1(2.8)	4(11.1)	9(25)	8(22.2)	18(50)	23(63.9)	0(0)	3(8.3)	12(33.3)	1(2.8)	14(38.9)	17(48.6)*
- C	0(0)	0(0)	0(0)	0(0)	1(2.8)	3(8.6)*	0(0)	0(0)	0(0)	0(0)	0(0)	2(5.6)	0(0)	0(0)	0(0)	0(0)	1(2.8)	2(5.7)*
Marginal adaptation																		
- A	36(100)	36(100)	36(100)	36(100)	30(83.3)	27(77.1)*	36(100)	36(100)	35(97.2)	34(94.4)	34(94.4)	26(72.2)	36(100)	35(97.2)	35(97.2)	36(100)	32(88.9)	26(74.3)*
- B	0 (0)	0 (0)	0 (0)	0(0)	6(16.7)	7(20)*	0(0)	0(0)	1(2.8)	2(5.6)	2(5.6)	9(25)	0(0)	1(2.8)	1(2.8)	0(0)	3(8.3)	8(22.9)*
- C	0 (0)	0 (0)	0 (0)	0(0)	0(0)	1(2.9)*	0(0)	0(0)	0(0)	0(0)	0(0)	1(2.8)	0(0)	0(0)	0(0)	0(0)	1(2.8)	1(2.9)*
Surface roughness																		
- A	36(100)	36(100)	36(100)	36(100)	36(100)	34(97.1)*	36(100)	36(100)	36(100)	36(100)	34(94.4)	32(88.9)	36(100)	36(100)	36(100)	36(100)	33(91.7)	30(85.7)*
- B	0(0)	0(0)	0(0)	36(100)	0(0)	1(2.9)*	0(0)	0(0)	0(0)	0(0)	2(5.6)	4(11.1)	0(0)	0(0)	0(0)	0(0)	2(5.6)	5(14.3)*
- C	0(0)	0(0)	0(0)	0(0)	0(0)	0(0)*	0(0)	0(0)	0(0)	0(0)	0(0)	0(0)	0(0)	0(0)	0(0)	0(0)	1(2.8)	0(0)*
Marginal discoloration																		
- A	35(97.2)	35(97.2)	35(97.2)	36 (100)	35(97.2)	34(97.1)*	9(25)	9(25)	6(16.7)	17(47.2)	14(38.9)	13(36.1)	24(66.7)	20(55.6)	14(38.9)	20(55.6)	19(52.8)	13(37.2)*
- B	1(2.8)	1(2.8)	1(2.8)	0(0)	1(2.8)	1(2.9)*	27(75)	27(75)	30(83.3)	19(52.8)	22(61.1)	23(63.9)	12(33.3)	16(44.4)	22(61.1)	16(44.4)	17(47.2)	22(62.8)*

* It has been calculated based on n=35

**Table 2:** Retention and caries outcomes of fissure sealants in MIH-affected molars over12 months

Groups	Sealant retention		p**	Caries development		Total	p**
	Successful	Unsuccessful		No	Yes		
	n (%)*	n (%)*		n (%)*	n (%)*	n (%)*	
Group 1	36 (100)	0 (0)	0.062	36 (100)	0 (0)	36 (100)	1.00
Group 2	32 (88.9)	4 (11.1)		35 (97.2)	1 (2.8)	36 (100)	
Group 3	36 (100)	0 (0)		35 (97.2)	1 (2.8)	36 (100)	
Group 4	34 (94.4)	2 (5.6)		36 (100)	0 (0)	36 (100)	
Group 5	36 (100)	0 (0)		36 (100)	0 (0)	36 (100)	
Group 6	33 (91.7)	3 (8.3)		35 (97.2)	1 (2.8)	36 (100)	
Total	207 (95.8)	9 (4.2)		213 (98.6)	3 (1.4)	216 (100)	

* Row percentages were calculated

** Fisher’s exact test

The plaque and gingival health status around the treated teeth were evaluated at baseline and at the 1st, 6th, and 12th months using the Silness & Löe Plaque Index and the Löe & Silness Gingival Index. Significant differences in both plaque and gingival index scores were observed over time (*p* < 0.005). For the plaque index, statistically significant differences were found between baseline and the 1st and 12th months, as well as between the 1st and 6th months (*p* < 0.005). For the gingival index, statistically significant differences were observed between baseline and the 1st month, and between baseline and the 12th months (*p* < 0.001). Significant differences were also observed between the 1st month and the 6th month (*p* < 0.001), as well as between the 1st and 12th months (*p* = 0.013). Both indices showed a decreasing trend over time, indicating reduced plaque accumulation and improved gingival health around the treated teeth.

Among the 216 teeth included in the study, 60.2% (*n* = 130) showed no air sensitivity, while 39.8% (*n* = 86) were sensitive. Comparison of air sensitivity scores revealed a statistically significant difference among the groups at baseline (*p* = 0.029). However, no significant differences were detected between the groups at any subsequent follow-up visit. Based on the generalized estimating equations (GEE) analysis, sensitivity scores differed significantly only at baseline (*p* = 0.018), with subsequent measurements at 1, 6, and 12 months showing no statistical significance (*p* = 0.727, 0.307, and 0.943, respectively). The test scores of all groups are presented in Table 3.

**Table 3:** Distribution and comparison of air sensitivity (SCASS) measurements of the study groups at baseline and controls

Groups	Descriptive statistics	Time			
		Baseline	1 month	6 months	12 months
Group 1	Median	1.00	0.00	0.00	0.00
	Mean ± SD	1.28 ± 0.66	0.17 ± 0.38	0.06 ± 0.23	0.00 ± 0.00
	Min-max	1.00-3.00	0.00-1.00	0.00-1.00	0.00-0.00
Group 2	Median	1.00	0.00	0.00	0.00
	Mean ± SD	1.71 ± 0.84	0.12 ± 0.33	0.00 ± 0.00	0.00 ± 0.00
	Min-max	1.00-3.00	0.00-1.00	0.00-0.00	0.00-0.00
Group 3	Median	1.00	0.00	0.00	0.00
	Mean ± SD	1.47 ±0.74	0.40 ± 0.50	0.07 ± 0.25	0.00 ± 0.00
	Min-max	1.00-3.00	0.00-1.00	0.00-1.00	0.00-0.00
Group 4	Median	1.00	0.00	0.00	0.00
	Mean ± SD	1.00 ± 0.00	0.20 ± 0.42	0.00 ± 0.00	0.00 ± 0.00
	Min-max	0.00-1.00	0.00-1.00	0.00-0.00	0.00-0.00
Group 5	Median	1.00	0.00	0.00	0.00
	Mean ± SD	1.13 ± 0.35	0.20 ± 0.41	0.13 ± 0.35	0.07 ± 0.25
	Min-max	2.00-1.00	1.00-0.00	1.00-0.00	1.00-0.00
Group 6	Median	1.00	0.00	0.00	0.00
	Mean ± SD	1.09 ± 0.30	0.27 ± 0.46	0.09 ± 0.30	0.00 ± 0.00
	Min-max	2.00-1.00	1.00-0.00	1.00-0.00	0.00-0.00
p*		0.029	0.502	0.652	0.467
p**		0.018	0.727	0.307	0.943

* Kruskal Wallis test

** Generalized Estimating Equations (GEE)

The distribution and comparison of marginal discoloration across the different groups and time points are summarized in Table 4. Statistically significant differences were observed in Groups 4, 5, and 6. In Group 4, a significant difference was found between the 1st and 12th months (*p* = 0.014). In Group 5, significant differences were detected between the 1st and 12th months (*p* < 0.001) and between the 6th and 12th months (*p* = 0.020). In Group 6, significant differences were observed between the 1st and 12th months (*p* = 0.003) and between the 6th and 12th months (*p* = 0.012).

**Table 4:** Distribution and comparison of marginal discoloration among groups and time points

Groups	Time (month)	Marginal Discoloration			p**
		Yes	no	Total	
		*n* (%)*	*n* (%)*	*n* (%)*	
Group 1	1	1 (2.8)	35 (97.2)	36 (100)	1.00
	6	1 (2.8)	35 (97.2)	36 (100)	
	12	1 (2.8)	35 (97.2)	36 (100)	
Group 2	1	0 (0)	36 (100)	36 (100)	0.368
	6	1 (2.8)	35 (97.2)	36 (100)	
	12	1 (2.9)	34 (97.1)	35 (100)	
Group 3	1	27 (75)	9 (25)	36 (100)	0.050
	6	27 (75)	9(25)	36 (100)	
	12	30 (83.3)	6 (16.7)	36 (100)	
Group 4	1	19_a_ (52.8)	17 _a_ (47.2)	36 (100)	0.039
	6	22_a, b_ (61.1)	14 _a, b_ (38.9)	36 (100)	
	12	23_b_ (63.9)	13_b_(36.1)	36 (100)	
Group 5	1	12 _a_ (33.3)	24_a_ (66.7)	36 (100)	0.001
	6	16 _a_ (44.4)	20_a_ (55.6)	36 (100)	
	12	22_b_ (61.1)	14_b_ (38.9)	36 (100)	
Group 6	1	16 _a_ (44.4)	20 _a_ (55.6)	36 (100)	0.006
	6	17_a_ (47.2)	19_a_ (52.8)	36 (100)	
	12	22_b_ (62.9)	13_b_ (37.1)	36 (100)	

* Row percentages were calculated

** Cochran's Q test

a-c Different lower script letters within each group indicate statistically significant differences between time points (Cochran’s Q test, followed by Bonferroni-corrected pairwise McNemar test; *p *< 0.05)

## Discussion

Hypomineralized enamel is characterized by increased porosity, reduced mineral content, and altered protein composition, which have been associated with compromised adhesion and higher rates of restoration and sealant failure compared with sound enamel [26, 27]. Hypothetically, modifying or stabilizing the hypomineralized enamel substrate prior to fissure sealing may influence clinical outcomes; however, the long-term effects of such approaches remain incompletely understood [17]. In the present study, only molars with occlusal involvement and at least one additional surface affected by MIH opacities were included, representing teeth at higher risk for post-eruptive breakdown and caries progression. Within this clinical context, biannual silver fluoride application was incorporated as an adjunctive preventive strategy to enhance enamel resistance and reduce hypersensitivity.

Previous literature indicates that the caries-arresting effect of a single SDF application may decline over time, and biannual reapplication has been recommended to sustain its therapeutic efficacy. Accordingly, repeated SF application was implemented across all groups to maximize caries prevention in this high-risk population [28, 29]. Following SF application, two fissure sealant materials were placed after using different application protocols, allowing comparisons based on both technique and material type.

Specifically, the clinical performance of glass ionomer and resin-based fissure sealants placed either during the same visit or one week following silver fluoride application was assessed in MIH-affected permanent molars of individuals at high risk for caries. Additionally, the study examined how different handling methods after SF application, such as rinsing or cotton drying during same-session procedures, influenced the clinical performance of the sealants.

The overall retention rate of sealants placed following SF application was high (95.8%), with no significant differences among the groups (*p* = 0.062). This finding indicates that the first null hypothesis, which proposed no differences in retention performance between groups, cannot be rejected. In practical terms, sealant retention over the 12-month follow-up was not significantly influenced by the timing of placement (immediate vs. one week after SF application), the type of material used (resin-based vs. glass ionomer) or the post SF handling protocol (rinsing vs. blot drying).

Previous clinical studies [30–32] evaluating the effect of application timing on the success of restorations placed immediately or after a delay following SDF application have also reported comparable outcomes. Consistent with the present results, a randomized controlled trial that compared single-visit and two-visit SMART protocols in asymptomatic primary molars reported no statistically significant difference in the clinical success of glass ionomer restorations between the groups [30].

Although the difference did not reach statistical significance, no loss of retention was detected at the 12-month follow-up in groups treated with resin-based fissure sealants, whereas each group receiving glass ionomer sealants exhibited retention loss in at least two teeth. The literature emphasizes that resin-based sealants provide superior long-term retention due to their micromechanical bonding and high marginal integrity achieved after polymerization. By contrast, while glass ionomers offer benefits such as chemical adhesion and fluoride release, they tend to show greater retention loss over time due to lower mechanical strength and reduced resistance to occlusal wear [33, 34].

The second null hypothesis, which proposed no difference in secondary caries development between the groups, was not rejected. In this study, fissure sealants demonstrated a very high caries-preventive effect in MIH-affected molars across all groups (98.6%), with no statistically significant differences detected. These results emphasize the significant protective effect of fissure sealants in preventing the development of new caries lesions in MIH-molars. Since hypersensitivity in MIH-affected molars has been shown to negatively influence toothbrushing behavior, thereby increasing caries risk, relieving hypersensitivity and protecting susceptible enamel surfaces with sealants may play an important role in caries prevention [35].

Taken together, these results indicate that applying sealants immediately after SF treatment is clinically acceptable and does not negatively affect short-term retention. The findings also show that SF pretreatment did not compromise adhesion under clinical conditions. However, laboratory studies report mixed results. While some suggest that SDF pretreatment has no effect on microleakage or may even enhance adhesion [36–38] others report a possible decrease in bond strength for both glass ionomer and resin-based restorations [39]. A recent systematic review attributed these discrepancies to methodological variations and material differences across studies [40]. Recent American Dental Association guidelines recommend 38% SDF as an option for nonrestorative caries management; however, they do not provide clear guidance on the optimal choice of restorative materials or the appropriate timing of their placement [41].

Air sensitivity, plaque accumulation and gingival health were also evaluated in this study. At baseline 39.8% of MIH-affected teeth exhibited air sensitivity, a rate higher than that was reported in previous studies [42]. This discrepancy may be attributed to differences in sample selection, evaluation criteria, and testing protocols. After treatment, both glass ionomer– and resin-based sealants placed following SF significantly reduced sensitivity, aligning with earlier studies that reported the desensitizing effects of SDF and the SMART approach in hypomineralized molars [16, 43]. In a previous study comparing silver fluoride (Riva Star Aqua) and glass ionomer–based fissure sealants in MIH-affected molars, 90% of children in both groups showed a notable reduction in sensitivity [44]. Similarly, combining fissure sealants with adhesive application or fluoride varnish have been recommended for hypersensitive MIH-molars [21, 45, 46]. The substantial decrease in post-treatment sensitivity observed in this study further reinforces the effectiveness of applying fissure sealants after SF treatment.

The plaque and gingival index values of the treated teeth decreased over time compared with baseline. Statistically significant difference was observed between baseline and the 1st and 12th months (*p* < 0.001). Although values at the 6th month were lower, the difference was not statistically significant. These findings suggest that oral hygiene motivation improved shortly after treatment but tended to decline over time. Nevertheless, regular follow-up visits appeared to help restore motivation, consistent with previous reports [47, 48]. The decrease in plaque and gingival index values around the treated teeth likely reflects enhanced local hygiene control resulting from post-treatment tooth-brushing education, sealing of retentive fissures, and relief of hypersensitivity.

A key drawback of using SDF is the discoloration that follows its application [49–52]. This staining is caused by the formation of silver compounds, including silver phosphate, silver chloride, and silver thiocyanate—after SDF application [53]. In the present study, discoloration was observed both at the restoration margins and on the tooth surfaces following treatment. When comparing restoration timing (immediate vs. one week after SF application), discoloration was markedly greater at all follow-up points for sealants placed immediately after SF. Conversely, significantly fewer discoloration cases occurred when sealants were applied one week later, with this difference reaching was statistical significance at all time points. Previous studies have also reported that delaying the placement of restorative materials after SDF application results in greater color stability compared to immediate restoration [32, 54]. The light sensitivity of silver ions has also been well documented, and it has been suggested that the light used during polymerization may contribute to dark discoloration [55]. This observation supports the hypothesis that light curing during immediate post-SDF restorations may intensify dark staining, whereas performing the restoration one week later allows the ions to stabilize and minimizing discoloration [32, 54, 55].

A key strength of this study is that it is the first to demonstrate that all treatment protocols following silver fluoride application effectively reduce hypersensitivity in MIH-affected molars, maintain fissure sealant adhesion, with no differences observed between groups in caries-related outcomes during the 12-month follow-up period. The absence of an early difference between immediate and delayed restorations after silver fluoride treatment may help alleviate the clinicians’ uncertainty about the best timing for restoration placement after silver fluoride application.

The limitations of the present study include the relatively small number of teeth per group, despite the overall large sample size, the limited duration of follow-up, the absence of lesion size assessment in MIH-affected teeth, and the uneven distribution of hypersensitive teeth across the groups. Also, the study did not include any patient reported outcome measures. In addition, the lack of a fissure sealant–only control group without silver fluoride application should be considered a limitation. This approach was deliberate, as the primary aim of the study was to compare different silver fluoride application protocols in terms of fissure sealant retention, rather than to assess the independent effect of fissure sealants alone. Future multicenter investigations with extended follow-up periods and inclusion of control groups without silver fluoride are needed to better determine the potential added benefit of silver fluoride in MIH-affected molars. Another limitation is that, while multiple teeth from the same patient were included, analyses were conducted at the tooth level without adjustment for within-patient clustering. The low number of outcome events limited the feasibility of applying more advanced clustered models such as multilevel or GEE-based analyses for categoric variables. As follow-up is ongoing, future analyses incorporating longer observation periods and potentially higher event rates will allow adjustment for within-patient clustering and provide more robust estimates.

It is important to emphasize that clinical decision-making in pediatric dentistry involves more than material performance alone. Factors such as chairside time, patient cooperation, and the practicality of completing treatment in a single visit are essential considerations. Although the use of silver fluoride represents an additional clinical step, it is a quick and minimally invasive procedure that can be performed in a short period of time without the need for local anesthesia. Within this context, the protocol combining silver fluoride application with subsequent fissure sealant placement was chosen to enhance preventive care in structurally compromised and porous MIH-affected teeth.

## Conclusion

Within the limitations of this prospective, randomized trial with a 12-month follow-up, both resin-based and glass ionomer fissure sealants placed after silver fluoride treatment demonstrated high clinical performance with comparable retention and no significant differences in caries-related outcomes between the groups. Although slight loss of retention was observed in some teeth with glass ionomer sealants, no statistically significant differences in retention were found between resin-based and glass ionomer sealants, irrespective of whether application occurred immediately or one week following silver fluoride treatment. Likewise, no significant differences were observed between the different post silver fluoride handling methods including cotton drying and rinsing. In addition to these primary outcomes, there was a marked reduction in air sensitivity along with clear improvements in plaque and gingival index scores over the follow-up period. These findings indicate that sealants placed after silver fluoride application not only effectively prevents caries but also enhances oral comfort and gingival health in children with MIH-affected molars.

## Data Availability

No datasets were generated or analysed during the current study.
