# Correction to: Effectiveness of fissure sealants following different silver fluoride application protocols in MIH-affected molars: randomized clinical study

**DOI:** 10.1007/s00784-026-06888-7

**Published:** 2026-04-27

**Authors:** Nagihan Cayiroglu, Elif Ballikaya, Gizem Erbas Unverdi, Zafer Cavit Cehreli

**Affiliations:** 1 Department of Pediatric Dentistry, Faculty of Dentistry, Hacettepe University, Ankara, 06100 Turkey; 2 Department of Pediatric Dentistry, Faculty of Dentistry, Istanbul Health and Technology University, Istanbul, Turkey

**Correction to: Clin Oral Invest**

10.1007/s00784-026-06856-1

In the published paper, the authors identified an error in the naming of the study groups in the Materials and Methods section. 

The group definitions were incorrectly reported. As presented in the tables and the main text, Groups 1, 3, and 5 correspond to the resin sealant groups. The corrected group names are provided below, and this correction has no impact on the analyses, results, or conclusions of the study.

The corrected group names are as follows:

Group 1: Resin-based fissure sealant (RBS; 3M™ Clinpro™ Sealant, 3 M ESPE, St. Paul, MN, USA), applied one week after SF application.

Group 2: Glass ionomer fissure sealant (GIS; GC Fuji TRIAGE®, GC Europe N.V., Leuven, Belgium), applied one week after SF application.

Group 3: RBS applied immediately after SF application following blot-drying.

Group 4: GIS applied immediately after SF application following blot-drying.

Group 5: RBS applied immediately after SF application following rinsing.

Group 6: GIS applied immediately after SF application following rinsing.

The original article has been corrected.

